# Prenylated Coumarins from *Heracleum stenopterum*, *Peucedanum praeruptorum*, *Clausena lansium*, and *Murraya paniculata*

**DOI:** 10.1007/s13659-016-0107-5

**Published:** 2016-09-19

**Authors:** Xiang-Mei Li, Xian-Jun Jiang, Ku Yang, Li-Xia Wang, Shi-Zhen Wen, Fei Wang

**Affiliations:** BioBioPha Co., Ltd., Kunming, 650201 People’s Republic of China

**Keywords:** *Heracleum stenopterum*, *Peucedanum praeruptorum*, *Clausena lansium*, *Murraya paniculata*, Prenylated coumarin, Cytotoxicity

## Abstract

**Abstract:**

Four hitherto unknown prenylated coumarins, namely 6″-*O*-*β*-d-apiofuranosylapterin (**1**), 4′-*O*-isobutyroylpeguangxienin (**2**), 6-(3-methyl-2-oxobutyroyl)-7-methoxycoumarin (**3**), and 6-hydroxycoumurrayin (**4**), were isolated from the ethanol extract of *Heracleum stenopterum*, *Peucedanum praeruptorum*, *Clausena lansium*, and *Murraya paniculata*, respectively. Their chemical structures were established on the basis of extensive spectroscopic analysis. Compound **2** exhibited in vitro cytotoxic activity against five human cancer cell lines (HL-60, A-549, SMMC-7721, MCF-7, and SW-480) with IC_50_ values ranging from 15.9 to 23.2 μM.

**Graphical Abstract:**

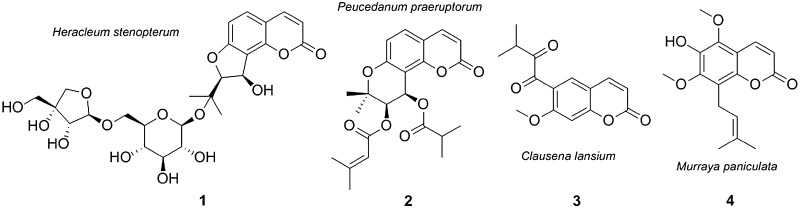

**Electronic supplementary material:**

The online version of this article (doi:10.1007/s13659-016-0107-5) contains supplementary material, which is available to authorized users.

## Introduction

**Fig. 1:**
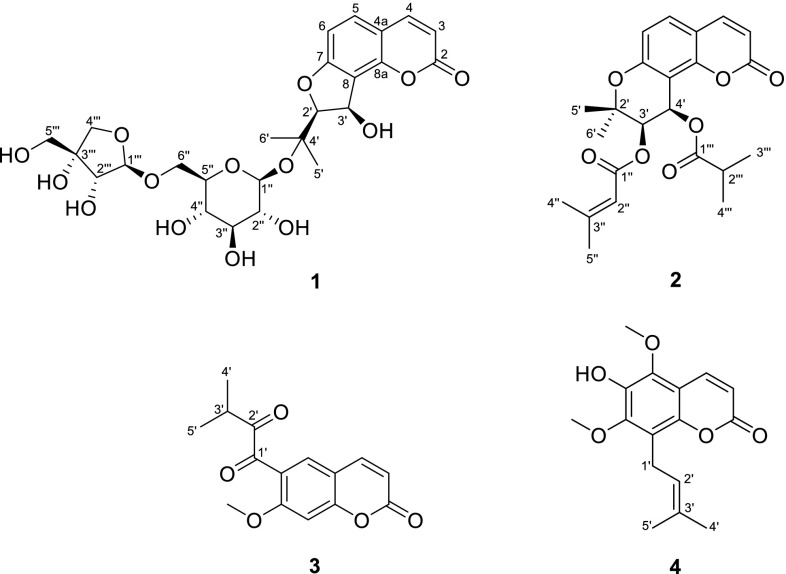
Structures of new coumarins **1**−**4**

Coumarins are typical secondary metabolites accumulated in the families Apiaceae (Umbelliferae) [1] and Rutaceae [2]. The widespread incorporation of prenyl units resulted in the structural diversity of coumarins in both families [2]. Particularly, the genera *Heracleum* and *Peucedanum* from Apiaceae can be characterized by the dominance of furanocoumarins [3] and pyranocoumarins [4], respectively. Coumarins possessed diverse pharmacological properties, in which the cytotoxic effects were most extensively examined [5]. As part of a BioBioPha (http://www.chemlib.cn/) objective to assemble a large-scale natural product library valuable in the discovery of new drug leads from nature [6, 7], four new prenylated coumarins Fig. 1, namely 6″-*O*-*β*-d-apiofuranosylapterin (**1**), 4′-*O*-isobutyroylpeguangxienin (**2**), 6-(3-methyl-2-oxobutyroyl)-7-methoxycoumarin (**3**), and 6-hydroxycoumurrayin (**4**), were isolated from the ethanol extract of *Heracleum stenopterum*, *Peucedanum praeruptorum*, *Clausena lansium*, and *Murraya paniculata*, respectively. This paper described the structure elucidation of new coumarins and their cytotoxicity evaluation against five human cancer cell lines.

## Results and Discussion

**Fig. 2:**
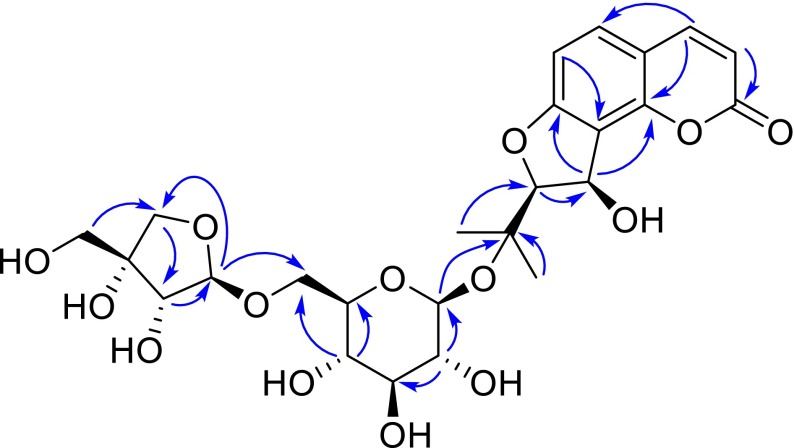
Key HMBC correlations of 6″-*O*-*β*-d-apiofuranosylapterin (**1**)

Compound **1**, obtained as white amorphous powder, possessed a molecular formula of C_25_H_32_O_14_, as evidenced by positive HRESIMS at *m*/*z* 579.1681 [M + Na]^+^ (calcd. for C_25_H_32_O_14_Na, 579.1684), requiring 10 degrees of unsaturation. The characteristic UV absorptions at 221 (sh), 248, 258, 325 nm indicated the presence of a 7-oxygenated coumarin chromophore [8]. The ^1^H NMR spectrum (Table 1) showed two pairs of aromatic doublets at *δ*_H_ 6.26 (d, *J* = 9.6 Hz, H-3), 8.00 (d, *J* = 9.6 Hz, H-4), 7.59 (d, *J* = 8.4 Hz, H-5), and 6.91 (d, *J* = 8.4 Hz, H-6), indicative of the existence of a 7,8-disubstituted coumarin moiety, two vicinal oxymethine protons of dihydrofuran ring at *δ*_H_ 4.51 (d, *J* = 6.4 Hz, H-2′) and 5.45 (dd, *J* = 8.8, 6.4 Hz, H-3′), and a *gem*-dimethyl group at *δ*_H_ 1.47 (s, H-5′, H-6′). The ^13^C NMR spectrum (Table 1) displayed a total of 25 carbon resonances, including nine coumarin carbons, and a set of carbons at *δ*_C_ 91.8 (d, C-2′), 68.2 (d, C-3′), 77.1 (s, C-4′), 24.6 (q, C-5′) and 23.0 (q, C-6′), which suggested that **1** should be an angular dihydrofuranocoumarin [9]. The remaining 11 oxygenated carbons were assignable to two sugar units. By comparison of its NMR spectra with published data [9, 10], an hexose was determined as a C-6 glycosylated glucopyranosyl [*δ*_H_ 4.55 (d, *J* = 7.8 Hz, H-1′′); *δ*_C_ 97.4 (d, C-1″), 73.3 (d, C-2″), 76.7 (d, C-3″), 69.7 (d, C-4″), 74.9 (d, C-5″) and 66.4 (t, C-6″)], and the pentose as a terminal apiofuranosyl moiety [*δ*_H_ 4.68 (d, *J* = 2.5 Hz, H-1″′); *δ*_C_ 109.0 (d, C-1‴), 75.9 (d, C-2‴), 78.8 (s, C-3‴), 73.3 (t, C-4‴), and 63.4 (t, C-5‴)]. In the HMBC spectrum (Fig. 2), the anomeric proton of glucose showed a strong correlation with C-4′, meanwhile, the anomeric one of apiose with the downfield shifted C-6″ (Δ*δ* ≈ +5.0 ppm) of glucose, which unambiguously established an api(1 → 6)glc sugar chain at C-4′. The same linkage pattern also occurred in the structure of heraclenol 3′-*O*-*β*-d-apiofuranosyl-(1 → 6)-*β*-d-glucopyranoside, isolated from this plant in our current research [11]. Based on comparison of the coupling constants (*cis*: ~6.0 Hz, *trans*: ~3.5 Hz) [12, 13], the *cis* configuration of H-2′/H-3′ was concluded. Therefore, compound **1** was identified as 6″-*O*-*β*-d-apiofuranosylapterin.

**Table 1:** NMR spectroscopic data for 6″-*O*-*β*-d-apiofuranosylapterin (**1**) in DMSO-*d*_6_ (*δ*_H_ 2.49, *δ*_C_ 39.5 ppm)

No.	*δ*_H_	*δ*_C_	No.	*δ*_H_	*δ*_C_
2		160.0 (s)	3″	3.13 (m)	76.7 (d)
3	6.26 (d, 9.6)	111.8 (d)	4″	3.02 (m)	69.7 (d)
4	8.00 (d, 9.6)	144.9 (d)	5″	3.13 (m)	74.9 (d)
4a		112.9 (s)	6″	3.21−3.27 (m)	66.4 (t)
5	7.59 (d, 8.4)	130.9 (d)	1‴	4.68 (d, 2.5)	109.0 (d)
6	6.91 (d, 8.4)	107.4 (d)	2‴	3.63 (dd, 6.3, 2.5)	75.9 (d)
7		162.8 (s)	3‴		78.8 (s)
8		116.8 (s)	4‴	3.72 (d, 9.4) 3.47 (d, 9.4)	73.3 (t)
8a		151.5 (s)	5‴	3.30 (dd, 11.5, 5.6) 3.26 (dd, 11.5, 5.6)	63.4 (t)
2′	4.51 (d, 6.4)	91.8 (d)	3′-OH	5.15 (d, 8.8)	
3′	5.45 (dd, 8.8, 6.4)	68.2 (d)	2″-OH	4.98 (d, 4.8)	
4′		77.1 (s)	3″-OH	4.94 (d, 5.0)	
5′	1.47 (s)	24.6 (q)	4″-OH	4.95 (d, 5.6)	
6′	1.47 (s)	23.0 (q)	2‴-OH	4.93 (d, 6.3)	
1″	4.55 (d, 7.8)	97.4 (d)	3‴-OH	4.41 (s)	
2″	2.89 (m)	73.3 (d)	5‴-OH	4.69 (t, 5.6)

Compound **2**, white amorphous powder, had a molecular formula of C_23_H_26_O_7_ by positive HRESIMS at *m/z* 437.1573 [M + Na]^+^ (calcd. for C_23_H_26_O_7_Na, 437.1571). The ^1^H NMR spectrum (Table 2) also indicated the existence of a 7,8-disubstituted coumarin moiety [*δ*_H_ 6.21 (d, *J* = 9.5 Hz, H-3), 7.59 (d, *J* = 9.5 Hz, H-4), 7.34 (d, *J* = 8.6 Hz, H-5), and 6.80 (d, *J* = 8.6 Hz, H-6)], two vicinal oxymethines bearing ester function at *δ*_H_ 5.36 (d, *J* = 5.0 Hz, H-3′) and 6.56 (d, *J* = 5.0 Hz, H-4′), a *gem*-dimethyl group at 1.44 (s, H-5′) and 1.40 (s, H-6′), a 3-methyl-2-butenoyl unit [*δ*_H_ 5.66 (s, H-2″), 2.16 (s, H-4″), 1.89 (s, H-5″)], and an isobutyroyl group [*δ*_H_ 2.57 (heptet, *J* = 7.0 Hz, H-2″′), 1.21 (d, *J* = 7.0 Hz, H-3‴), and 1.17 (d, *J* = 7.0 Hz, H-4‴)]. The ^13^C NMR spectrum (Table 2) exhibited a total of 23 carbon resonances, including a set of signals due to a dihydropyran ring [*δ*_C_ 77.7 (s, C-2′), 68.7 (d, C-3′), 60.8 (d, C-4′), 22.9 (q, C-5′) and 24.5 (q, C-6′)], as well as two ester carbonyl carbons [*δ*_C_ 165.2 (s, C-1″) and 175.7 (s, C-1‴)]. Comparison of its NMR data with those of hyuganin D [14], also isolated in our current research, revealed that **2** was an analogue of the angular dihydropyranocoumarin. The obvious difference was that a 3-methyl-2-butenoyl moiety replaced the acetyl group. The acylated positions were determined by the HMBC correlations from H-3′ to C-1″ of 3-methyl-2-butenoyl group, and from H-4′ to C-1‴ of isobutyroyl group. The *cis* configuration of H-3′ and H-4′ was concluded based on the diagnostic coupling constant [15]. From the above results, the structure of compound **2** was established as 4′-*O*-isobutyroylpeguangxienin.

**Table 2:** NMR spectroscopic data for 4′-*O*-isobutyroylpeguangxienin (**2**) in CDCl_3_ (*δ*_H_ 7.26, *δ*_C_ 77.0 ppm)

No.	*δ*_H_	*δ*_C_	No.	*δ*_H_	*δ*_C_
2		159.8 (s)	5′	1.44 (s)	22.9 (q)
3	6.21 (d, 9.5)	113.1 (d)	6′	1.40 (s)	24.5 (q)
4	7.59 (d, 9.5)	143.3 (d)	1″		165.2 (s)
4a		112.5 (s)	2″	5.66 (s)	115.0 (d)
5	7.34 (d, 8.6)	129.0 (d)	3″		158.9 (s)
6	6.80 (d, 8.6)	114.4 (d)	4″	2.16 (s)	20.3 (q)
7		156.8 (s)	5″	1.89 (s)	27.5 (q)
8		107.2 (s)	1″′		175.7 (s)
8a		154.0 (s)	2″′	2.57 (heptet, 7.0)	34.1 (d)
2′		77.7 (s)	3″′	1.21 (d, 7.0)	18.7 (q)
3′	5.36 (d, 5.0)	68.7 (d)	4″′	1.17 (d, 7.0)	18.8 (q)
4′	6.56 (d, 5.0)	60.8 (d)			

Compound **3**, yellow amorphous powder, possessed a molecular formula of C_15_H_14_O_5_ according to the positive HRESIMS at *m/z* 297.0737 [M + Na]^+^ (calcd. for C_15_H_14_O_5_Na, 297.0733). The NMR data (Table 3) revealed the presence of a 6,7-disubstituted coumarin skeleton [*δ*_H_ 6.33 (d, *J* = 9.6 Hz, H-3), 7.69 (d, *J* = 9.6 Hz, H-4), 7.97 (s, H-5), and 6.83 (s, H-8)], a methoxy group (*δ*_H_ 3.89; *δ*_C_ 56.5), and a set of signals originated from a prenyl unit [*δ*_H_ 3.15 (heptet, *J* = 7.0, H-3′), 1.24 (d, *J* = 7.0, H-4′ and H-5′); *δ*_C_ 193.6 (s, C-1′), 205.6 (s, C-2′), 36.3 (d, C-3′), 17.2 (q, C-4′ and C-5′)], which were further established as a 3-methyl-2-oxobutyroyl unit by the HMBC correlations of H-4′ and H-5′ with C-2′ as well as comparison with literature data [16]. This unit was attached to C-6 from the HMBC correlation of H-5 to C-1′, while the methoxy at C-7 by a weak but clear ^4^ *J* correlation from the methoxy protons to C-8. Accordingly, compound **3** was determined as 6-(3-methyl-2-oxobutyroyl)-7-methoxycoumarin.

**Table 3:** NMR spectroscopic data for 6-(3-methyl-2-oxobutyroyl)-7-methoxycoumarin (**3**) and 6-hydroxycoumurrayin (**4**) in CDCl_3_ (*δ*_H_ 7.26, *δ*_C_ 77.0 ppm)

No.	**3**		**4**	
	*δ*_H_	*δ*_C_	*δ*_H_	*δ*_C_
2		159.7 (s)		161.2 (s)
3	6.33 (d, 9.6)	114.6 (d)	6.33 (d, 9.6)	114.5 (d)
4	7.69 (d, 9.6)	143.0 (d)	7.92 (d, 9.6)	138.3 (d)
4a		113.0 (s)		110.0 (s)
5	7.97 (s)	131.1 (d)		140.9 (s)
6		120.9 (s)		138.4 (s)
7		162.2 (s)		149.4 (s)
8	6.83 (s)	100.1 (d)		118.9 (s)
8a		159.3 (s)		146.0 (s)
1′		193.6 (s)	3.50 (d, 7.0)	22.5 (t)
2′		205.6 (s)	5.22 (br t, 7.0)	121.3 (d)
3′	3.15 (heptet, 7.0)	36.3 (d)		132.8 (s)
4′	1.24 (d, 7.0)	17.2 (q)	1.68 (br s)	25.7 (q)
5′	1.24 (d, 7.0)	17.2 (q)	1.83 (br s)	18.0 (q)
5-OCH_3_			3.96 (s)	62.1 (q)
6-OH			5.56 (s)	
7-OCH_3_	3.89 (s)	56.5 (q)	3.91 (s)	61.7 (q)

Compound **4**, white amorphous powder, had a molecular formula of C_16_H_18_O_5_ determined by positive HRESIMS at *m/z* 313.1042 [M + Na]^+^ (calcd. for C_16_H_18_O_5_Na, 313.1046). The NMR spectra (Table 3) showed the presence of a 5,6,7,8-tetrasubstituted coumarin skeleton [*δ*_H_ 6.33 (d, *J* = 9.6 Hz, H-3) and 7.92 (d, *J* = 9.6 Hz, H-4); *δ*_C_ 161.2 (s, C-2), 114.5 (d, C-3), 138.3 (d, C-4), 140.9 (s, C-5), 138.4 (s, C-6), 149.4 (s, C-7), 118.9 (s, C-8), 110.0 (s, C-4a), and 146.0 (s, C-8a)], two methoxys [*δ*_H_ 3.96, 3.91 (each s); *δ*_C_ 62.1, 61.7 (each q)], a prenyl [*δ*_H_ 3.50 (d, *J* = 7.0 Hz, H-1′), 5.22 (br t, *J* = 7.0, H-2′), 1.68 (br s, H-4′), and 1.83 (br s, H-5′); *δ*_C_ 22.5 (t, C-1′), 121.3 (d, C-2′), 132.8 (s, C-3′), 25.7 (q, C-4′), 18.0 (q, C-5′)], and a phenolic hydroxy group [*δ*_H_ 5.56 (s, 6-OH)]. All of the above spectroscopic data were generally consistent with those of 6-methoxycoumurrayin [16], except that a methoxy group was replaced by a hydroxy group. The two methoxy groups were located at C-5 and C-7 on the basis of the HMBC correlations from H-4 to C-5, 5-OCH_3_ to C-5, H-1′ to C-7/C-8a, and from 7-OCH_3_ to C-7. Thus, compound **4** was identified as 6-hydroxycoumurrayin.

The in vitro cytotoxicity of these new coumarins (**1**–**4**) was evaluated against five human cancer cell lines (HL-60, A-549, SMMC-7721, MCF-7, and SW-480) using the MTS method. DDP (cisplatin) and paclitaxel were used as positive controls. Compound **2** exhibited cytotoxic activity with IC_50_ values ranging from 15.9 to 23.2 μM for all tested cell lines, while the other compounds were inactive (IC_50_ values >40 μM).

## Experimental Section

### General Experimental Procedures

Optical rotations were measured on a Jasco P-1020 automatic digital polarimeter. UV data were obtained from HPLC online analysis. NMR spectra were carried out on a Bruker AV-400, DRX-500, Avance III 600, or AV-800 spectrometer with deuterated solvent signals used as internal standards. ESI and HRESIMS were performed with a Shimadzu LC-IT-TOF mass spectrometer equipped with an ESI interface (Shimadzu, Kyoto, Japan). Silica gel 200–300 mesh (Qingdao Marine Chemical Inc., Qingdao, China), Chromatorex C-18 (40–75 μm, Fuji Silysia Chemical Ltd., Japan) and Sephadex LH-20 (GE Healthcare Bio-Sciences AB, Uppsala, Sweden) were used for normal pressure column chromatography. Prep-HPLC separation was performed using an Agilent 1260 series HPLC system equipped with a Zorbax SB-C18 column (5 μm, 21.2 × 150 mm). Fractions were monitored and analyzed by TLC, in combination with an Agilent 1200 series HPLC system equipped by an Extend-C18 column (5 μm, 4.6 × 150 mm).

### Plant Material and Isolation (See Table 4)

The retention times (*t*_R_) of **1**–**4** on an analytical HPLC Extend-C18 column (20 → 100 % MeOH in H_2_O over 8.0 min followed by 100 % MeOH to 13.0 min, 1.0 ml/min, 25 °C) were 5.86, 9.69, 7.83, and 8.77 min, respectively.

**Table 4:** Plant material and isolation

	Family	Species	Plant parts	Place of origin	Plant wt. (kg)	Extract wt. (g)	Compd. wt. (mg)
**1**	Apiaceae	*H*. *stenopterum*	Whole plant	Yunnan, CN	5.5	460	20
**2**	Apiaceae	*P*. *praeruptorum*	Roots	Sichuan, CN	10.0	1300	234
**3**	Rutaceae	*C*. *lansium*	Twigs and leaves	Yunnan, CN	12.0	200	201
**4**	Rutaceae	*M*. *paniculata*	Twigs and leaves	Yunnan, CN	8.5	700	23

### 6″-*O*-*β*-d-Apiofuranosylapterin (**1**)

White amorphous powder; UV (MeOH) *λ*_max_: 221 (sh), 248, 258, 325 nm; $\left[ \alpha \right]_{\text{D}}^{25}$ +114.7 (*c* 0.20, MeOH); ^1^H NMR and ^13^C NMR data: see Table 1; ESIMS (pos.): *m/z* 579 [M + Na]^+^; HRESIMS (pos.): *m/z* 579.1681 [M + Na]^+^ (calcd. for C_25_H_32_O_14_Na, 579.1684).

### 4′-*O*-Isobutyroylpeguangxienin (**2**)

White amorphous powder; UV (MeOH) *λ*_max_: 218, 256 (sh), 296 (sh), 323 nm; $\left[ \alpha \right]_{\text{D}}^{25}$ +38.8 (*c* 0.20, MeOH); ^1^H NMR and ^13^C NMR data: see Table 2; ESIMS (pos.): *m/z* 437 [M + Na]^+^; HRESIMS (pos.): *m/z* 437.1573 [M + Na]^+^ (calcd. for C_23_H_26_O_7_Na, 437.1571).

### 6-(3-Methyl-2-oxobutyroyl)-7-methoxycoumarin (**3**)

Yellow amorphous powder; UV (MeOH) *λ*_max_: 215 (sh), 227 (sh), 261, 308, 340 nm; ^1^H NMR and ^13^C NMR data: see Table 3; ESIMS (pos.): *m/z* 297 [M + Na]^+^; HRESIMS (pos.): *m/z* 297.0737 [M + Na]^+^ (calcd. for C_15_H_14_O_5_Na, 297.0733).

### 6-Hydroxycoumurrayin (**4**)

White amorphous powder; UV (MeOH) *λ*_max_: 232 (sh), 307, 357 (sh) nm; ^1^H NMR and ^13^C NMR data: see Table 3; ESIMS (pos.): *m/z* 313 [M + Na]^+^; HRESIMS (pos.): *m/z* 313.1042 [M + Na]^+^ (calcd. for C_16_H_18_O_5_Na, 313.1046).

### Cytotoxicity Assays

Five human tumor cell lines (HL-60, A-549, SMMC-7721, MCF-7, and SW-480) obtained from ATCC (Manassas, VA, USA) were used in the cytotoxicity assay. All cells were cultured in RPMI-1640 or DMEM medium (Hyclone, Logan, UT, USA), supplemented with 10 % fetal bovine serum (Hyclone) at 37 °C in a humidified atmosphere containing 5 % CO_2_. Cell viability was assessed by conducting colorimetric measurements of the amount of insoluble formazan formed in living cells based on the reduction of MTS (Sigma, St. Louis, MO, USA). Briefly, 100 μL of adherent cells were seeded into each well of a 96-well cell culture plate and allowed to adhere for 12 h before drug addition, while suspended cells were seeded just before drug addition, both with an initial density of 1 × 10^5^ cells/mL in 100 μL medium. Each cell line was exposed to the test compound at various concentrations in triplicate for 48 h, with cisplatin and paclitaxel as positive controls. After the incubation, 20 μL MTS and 100 μL medium was added to each well after removal of 100 μL medium, and the incubation continued for 2–4 h at 37 °C. The optical density was measured at 492 nm using a Multiskan FC plate reader (Thermo Scientific, USA). The IC_50_ value of each compound was calculated according to the Reed and Muench method.

## Electronic supplementary material

Below is the link to the electronic supplementary material.

Supplementary material 1 (DOCX 541 kb)
