# Supplementary material for: Prenylated Coumarins from Heracleum stenopterum, Peucedanum praeruptorum, Clausena lansium, and Murraya paniculata
Source: Nat Prod Bioprospect. 2016 Sep 19;6(5):233–7. doi: 10.1007/s13659-016-0107-5 (PMC5080209; doi:10.1007/s13659-016-0107-5)

**Electronic Supplementary Material**

**Prenylated coumarins from *Heracleum stenopterum*, *Peucedanum praeruptorum*, *Clausena lansium*, and *Murraya paniculata***

Xiang-Mei Li • Xian-Jun Jiang • Ku Yang • Li-Xia Wang • Shi-Zhen Wen • Fei Wang

**Electronic supplementary material** The online version of this article (doi:#####) contains supplementary material, which is available to authorized users.

X.-M. Li • X.-J. Jiang • K. Yang • L.-X. Wang • S.-Z. Wen • F. Wang (🖂)

BioBioPha Co., Ltd., Kunming 650201, People’s Republic of China

e-mail: f.wang@mail.biobiopha.com

**Content list:**

**S1.** ^1^H NMR spectrum (800 MHz, DMSO-*d*_6_) of 6′′-*O*-*β*-D-apiofuranosylapterin (**1**)

**S2.** ^13^C NMR spectrum (200 MHz, DMSO-*d*_6_) of 6′′-*O*-*β*-D-apiofuranosylapterin (**1**)

**S3.** HSQC spectrum (800 MHz, DMSO-*d*_6_) of 6′′-*O*-*β*-D-apiofuranosylapterin (**1**)

**S4.** HMBC spectrum (500 MHz, DMSO-*d*_6_) of 6′′-*O*-*β*-D-apiofuranosylapterin (**1**)

**S5.** ^1^H NMR spectrum (500 MHz, CDCl_3_) of 4′-*O*-isobutyroylpeguangxienin (**2**)

**S6.** ^13^C NMR spectrum (100 MHz, CDCl_3_) of 4′-*O*-isobutyroylpeguangxienin (**2**)

**S7.** HMBC spectrum (500 MHz, CDCl_3_) of 4′-*O*-isobutyroylpeguangxienin (**2**)

**S8.** ^1^H NMR spectrum (400 MHz, CDCl_3_) of 6-(3-methyl-2-oxobutyroyl)-7-methoxycoumarin (**3**)

**S9.** ^13^C NMR spectrum (100 MHz, CDCl_3_) of 6-(3-methyl-2-oxobutyroyl)-7-methoxycoumarin (**3**)

**S10.** HMBC spectrum (500 MHz, CDCl_3_) of 6-(3-methyl-2-oxobutyroyl)-7-methoxycoumarin (**3**)

**S11.** ^1^H NMR spectrum (400 MHz, CDCl_3_) of 6-hydroxycoumurrayin (**4**)

**S12.** ^13^C NMR spectrum (150 MHz, CDCl_3_) of 6-hydroxycoumurrayin (**4**)

**S13.** HMBC spectrum (600 MHz, CDCl_3_) of 6-hydroxycoumurrayin (**4**)

**S1.** ^1^H NMR spectrum (800 MHz, DMSO-*d*_6_) of 6′′-*O*-*β*-D-apiofuranosylapterin (**1**)

**
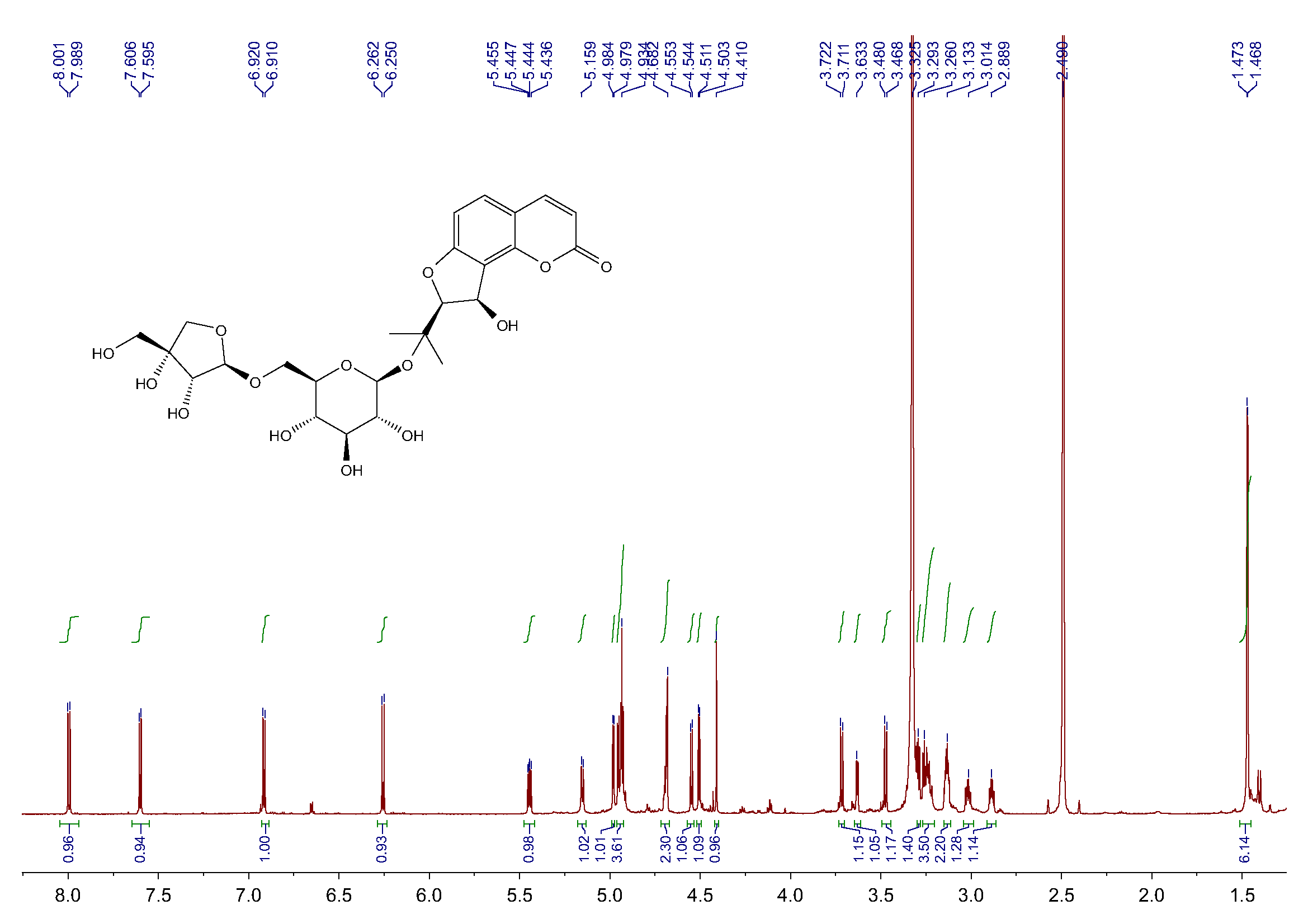
**

**S2.** ^13^C NMR spectrum (200 MHz, DMSO-*d*_6_) of 6′′-*O*-*β*-D-apiofuranosylapterin (**1**)

**
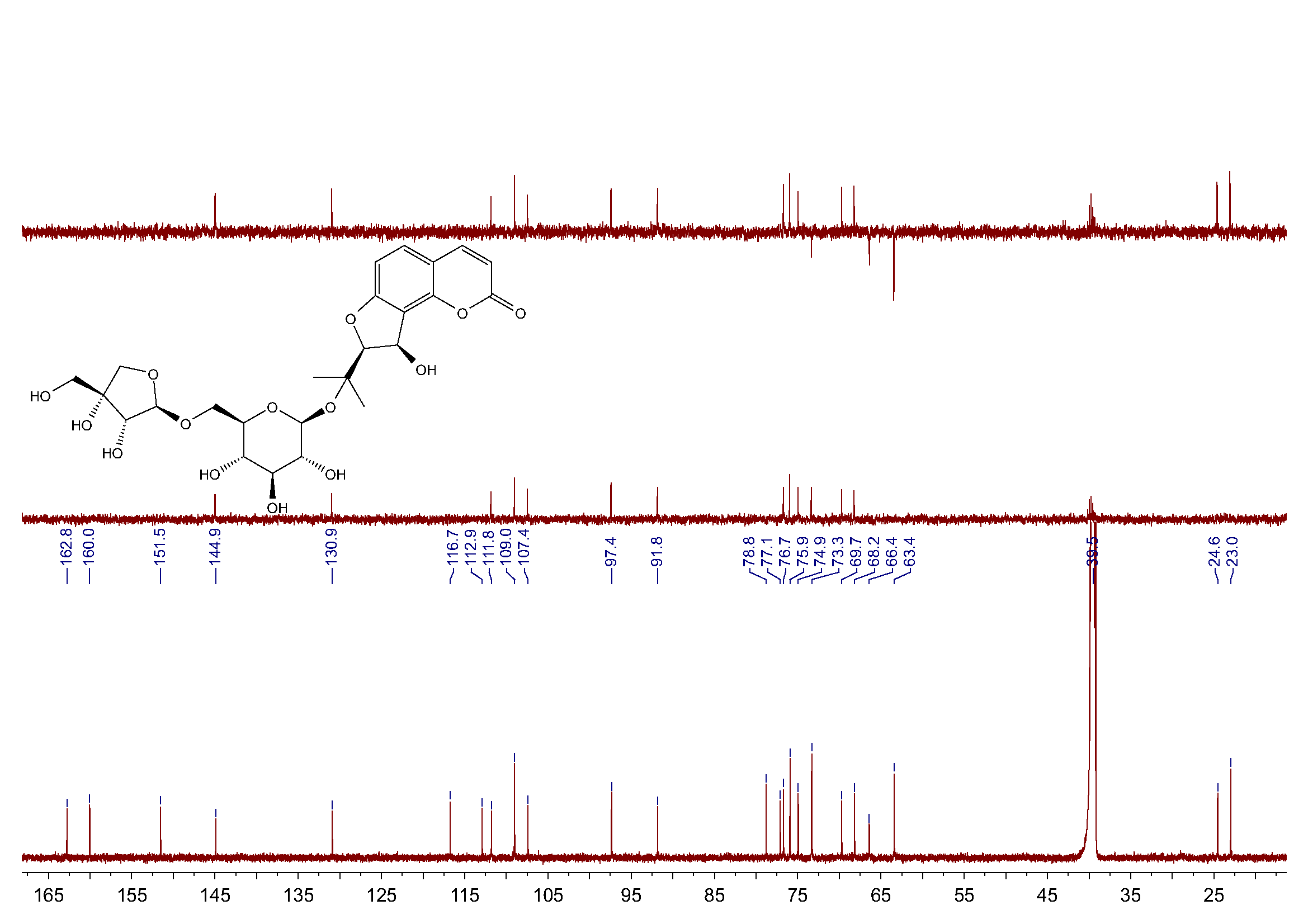
**

**S3.** HSQC spectrum (800 MHz, DMSO-*d*_6_) of 6′′-*O*-*β*-D-apiofuranosylapterin (**1**)

**
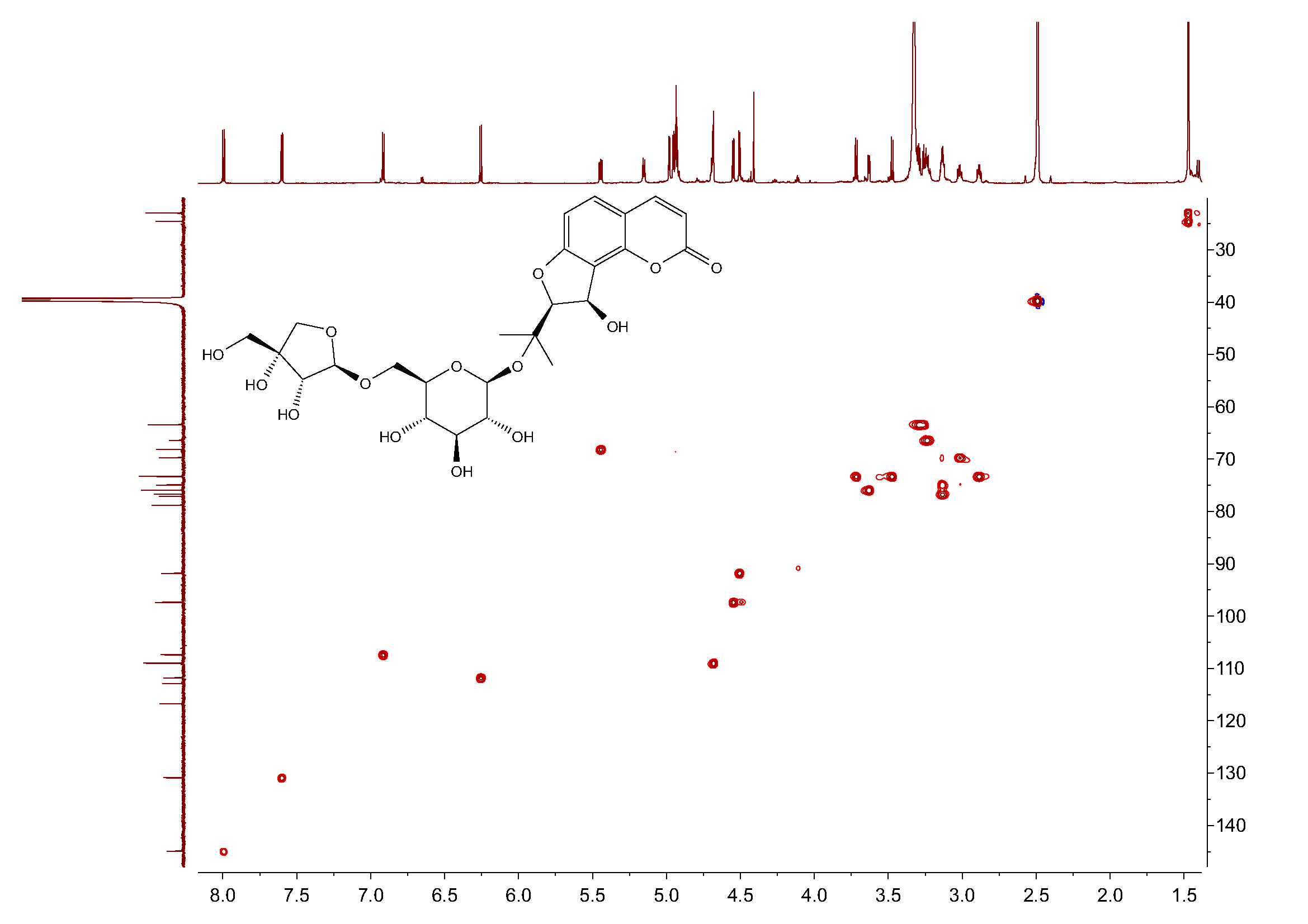
**

**S4.** HMBC spectrum (500 MHz, DMSO-*d*_6_) of 6′′-*O*-*β*-D-apiofuranosylapterin (**1**)

**
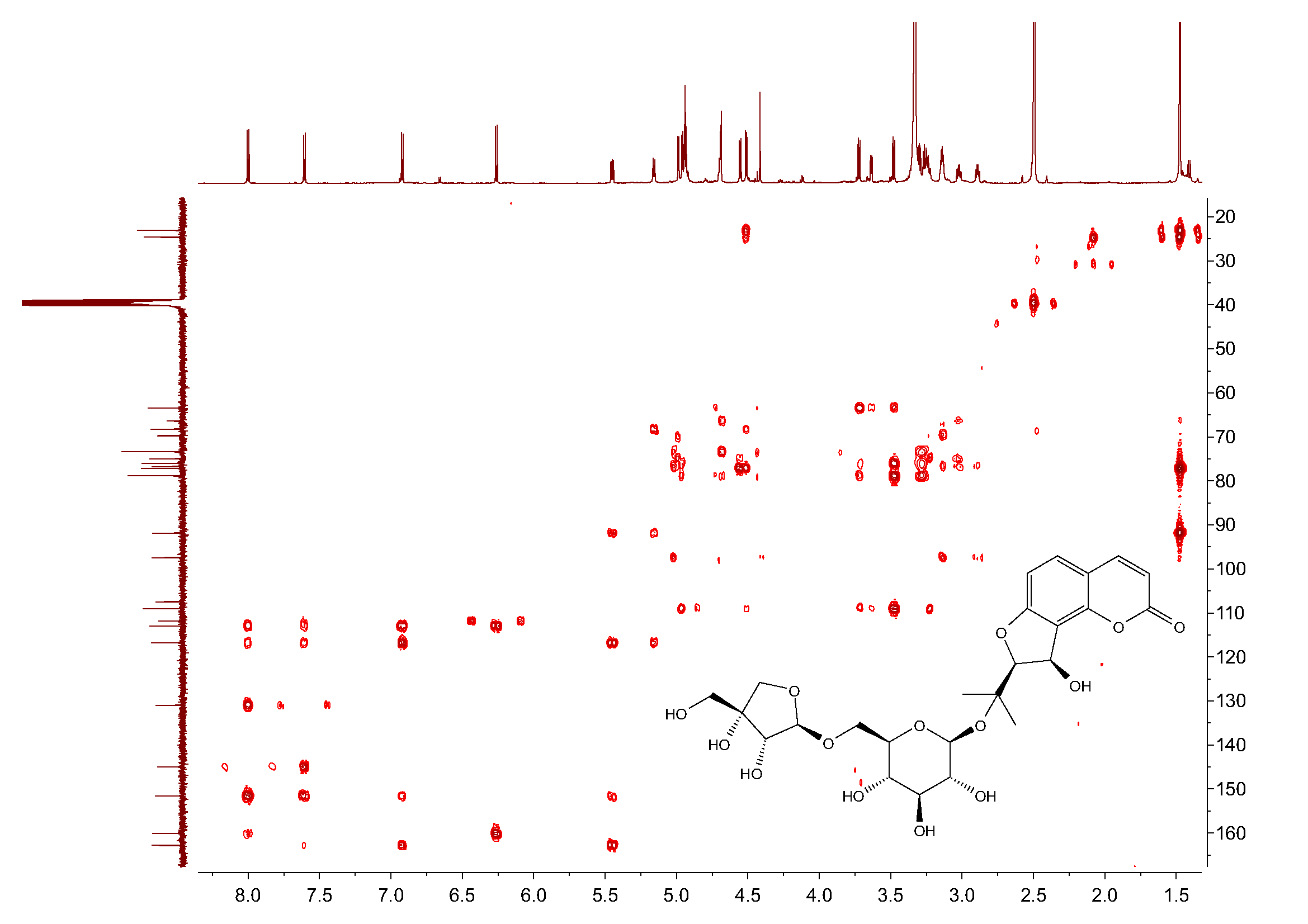
**

**S5.** ^1^H NMR spectrum (500 MHz, CDCl_3_) of 4′-*O*-isobutyroylpeguangxienin (**2**)


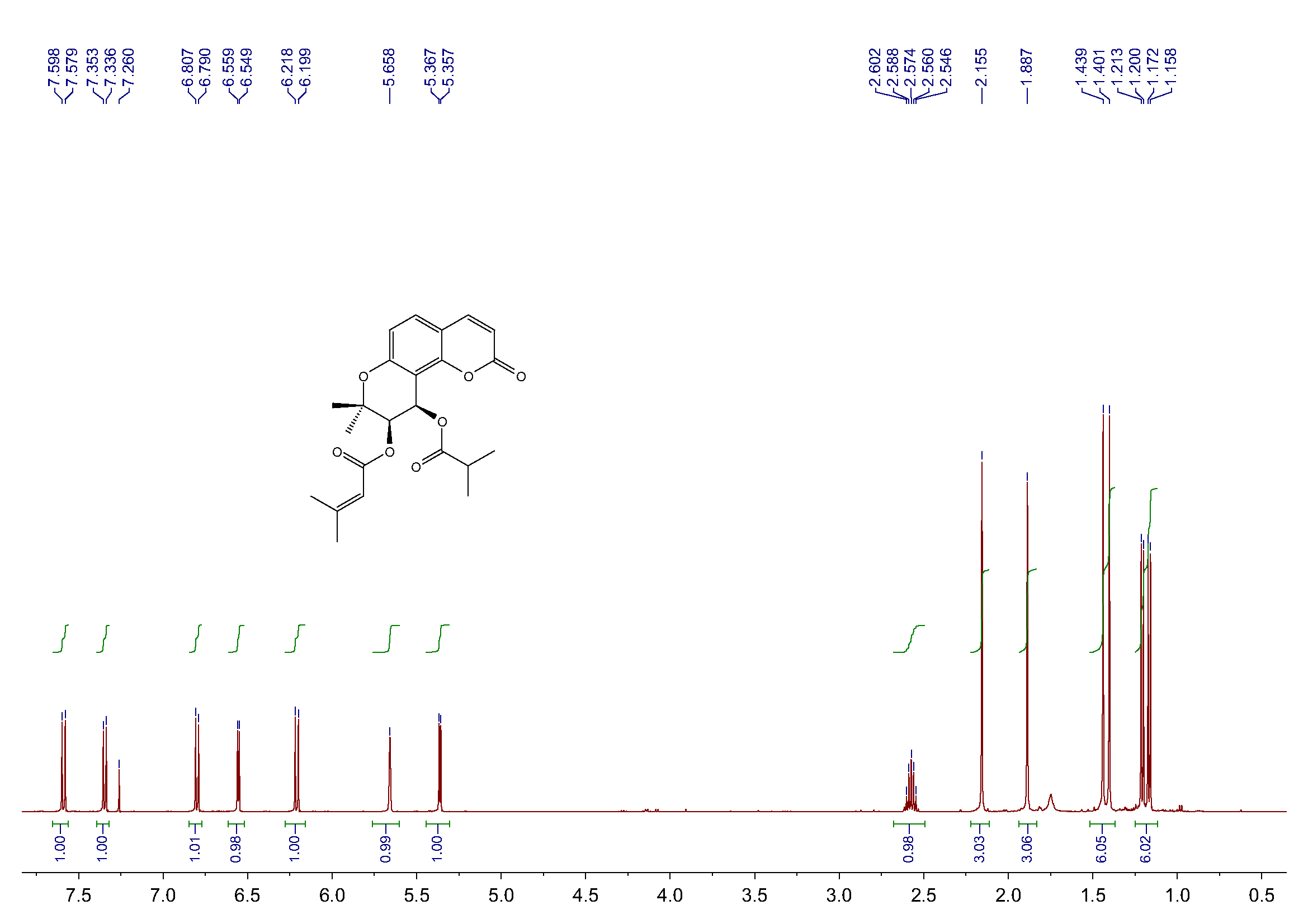


**S6.** ^13^C NMR spectrum (100 MHz, CDCl_3_) of 4′-*O*-isobutyroylpeguangxienin (**2**)


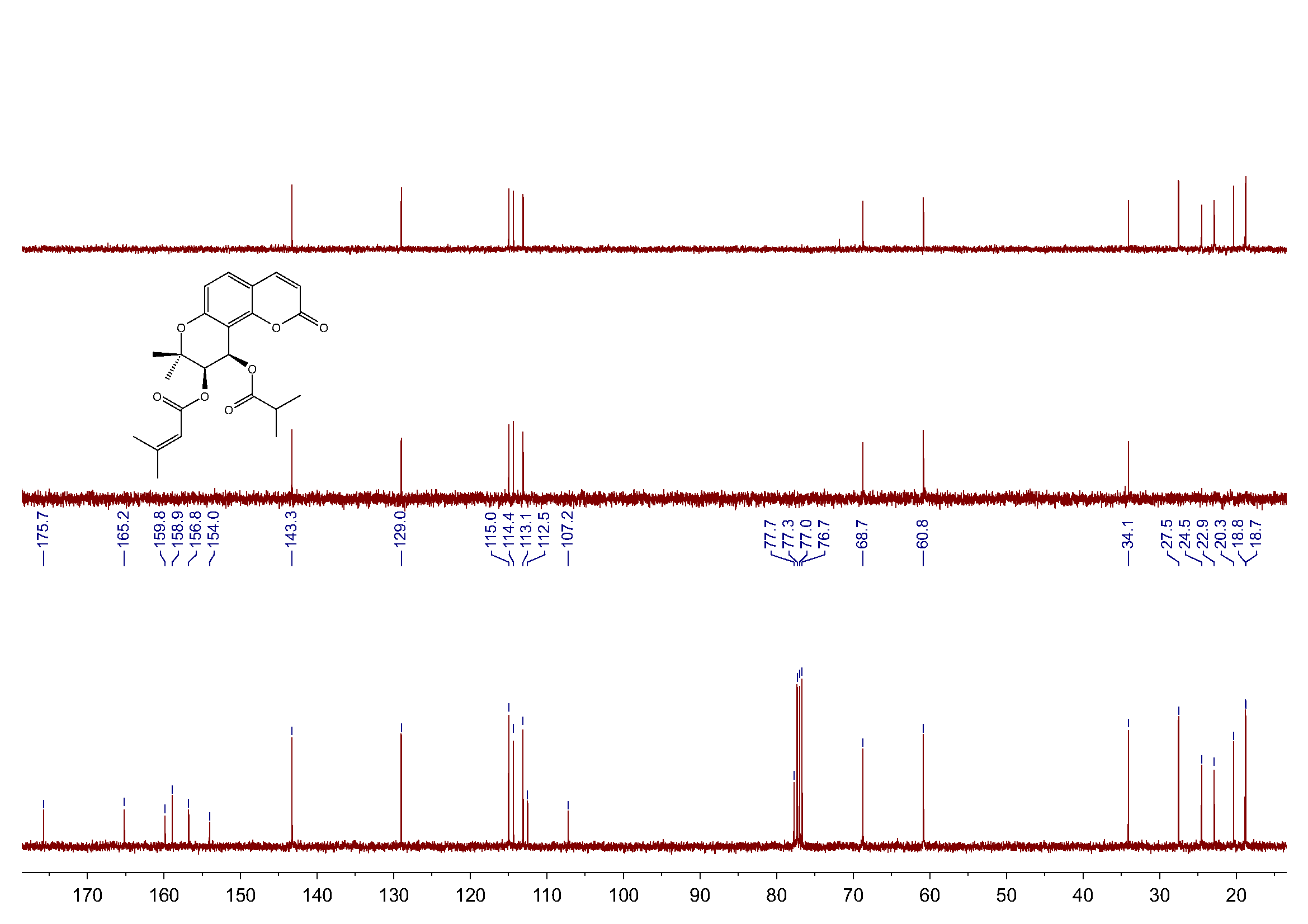


**S7.** HMBC spectrum (500 MHz, CDCl_3_) of 4′-*O*-isobutyroylpeguangxienin (**2**)


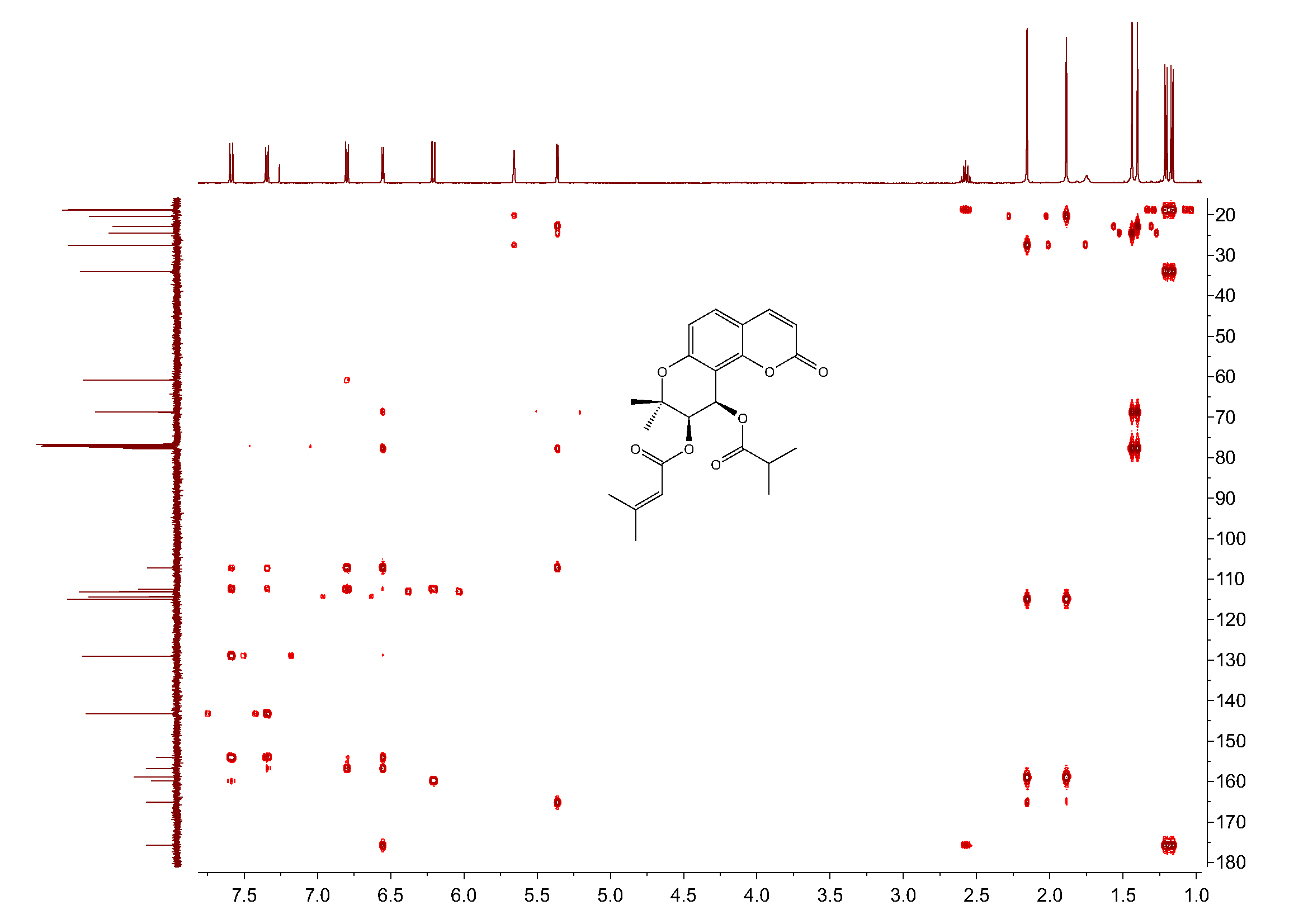


**S8.** ^1^H NMR spectrum (400 MHz, CDCl_3_) of 6-(3-methyl-2-oxobutyroyl)-7-methoxycoumarin (**3**)


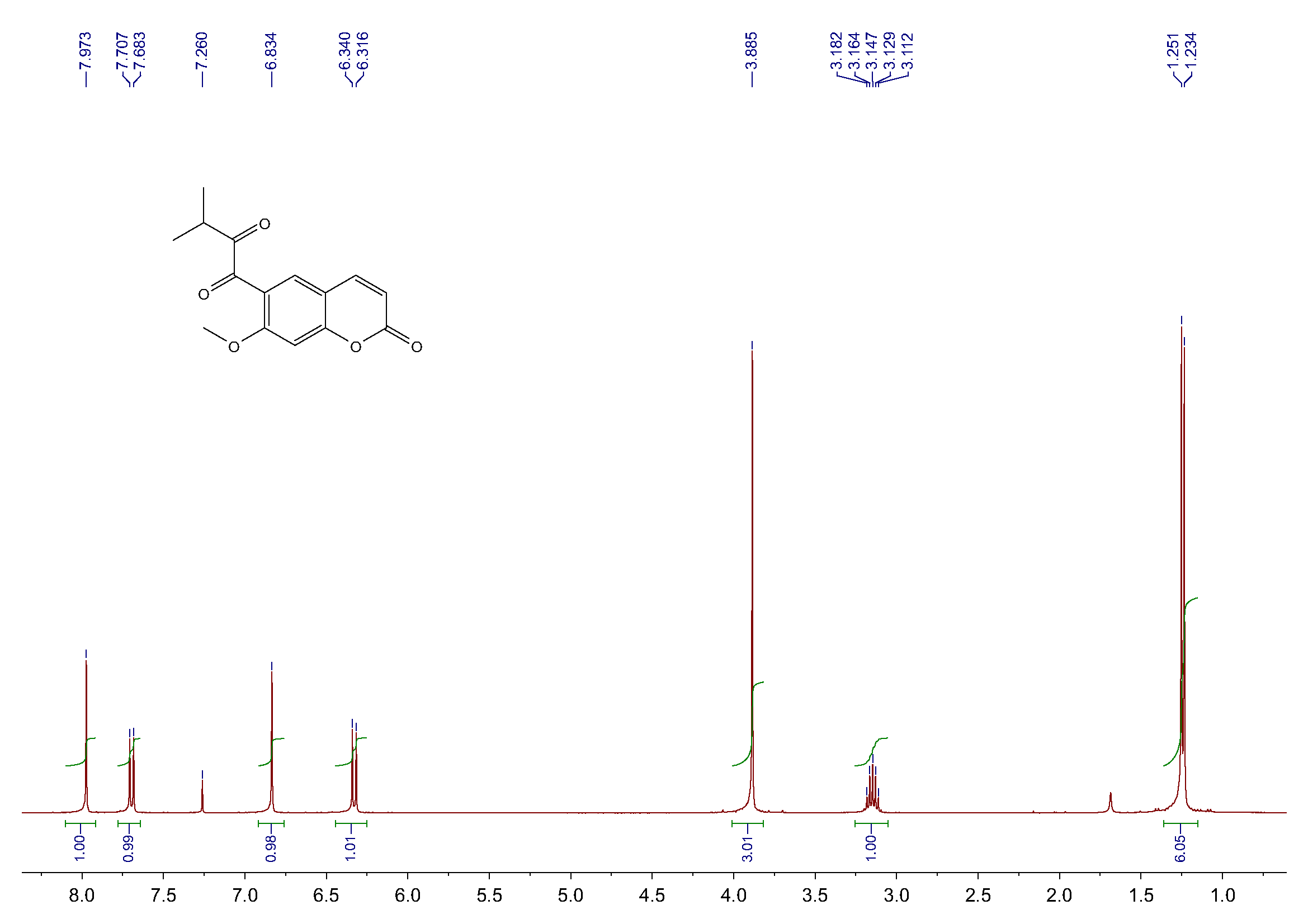


**S9.** ^13^C NMR spectrum (100 MHz, CDCl_3_) of 6-(3-methyl-2-oxobutyroyl)-7-methoxycoumarin (**3**)


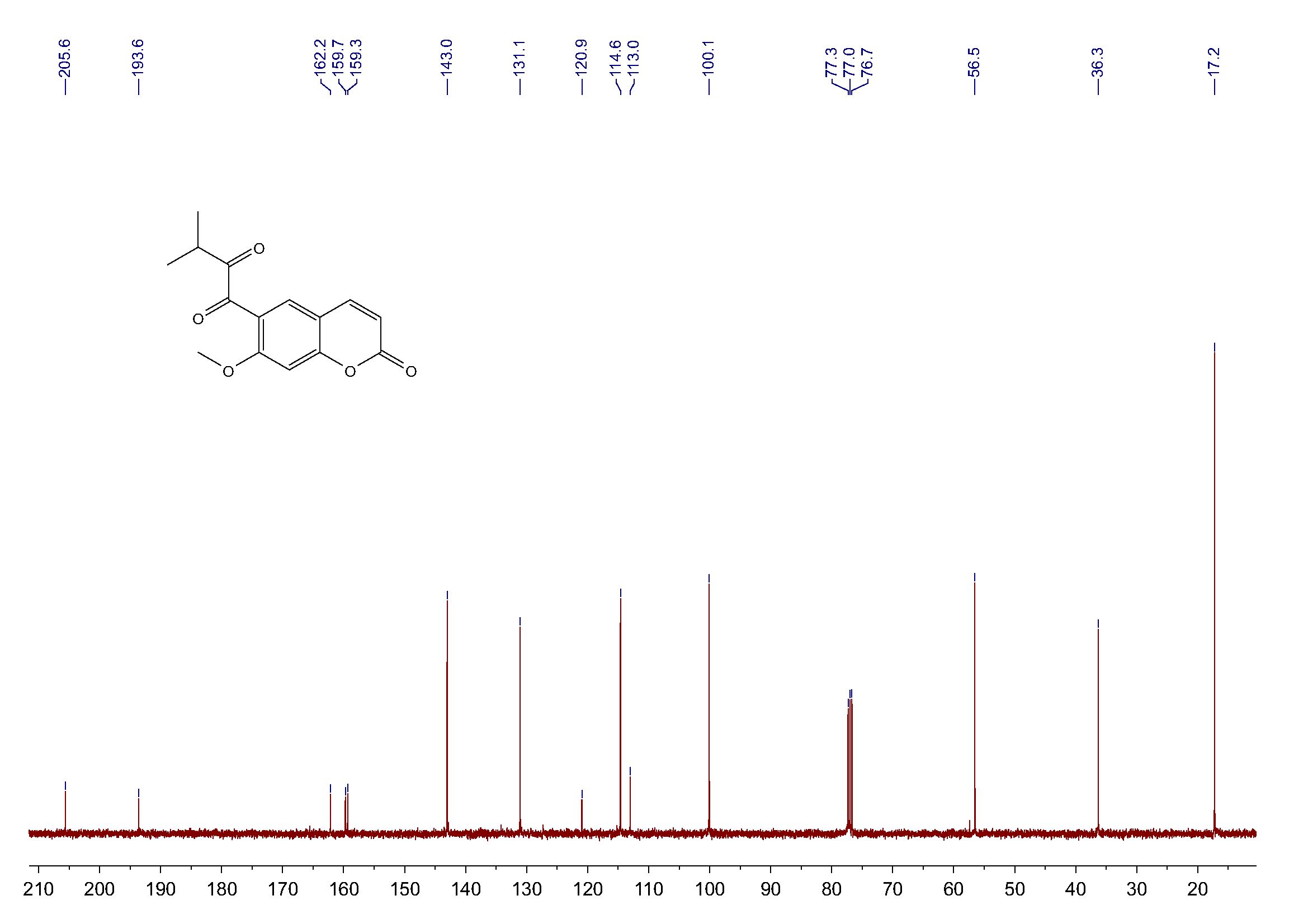


**S10.** HMBC spectrum (500 MHz, CDCl_3_) of 6-(3-methyl-2-oxobutyroyl)-7-methoxycoumarin (**3**)


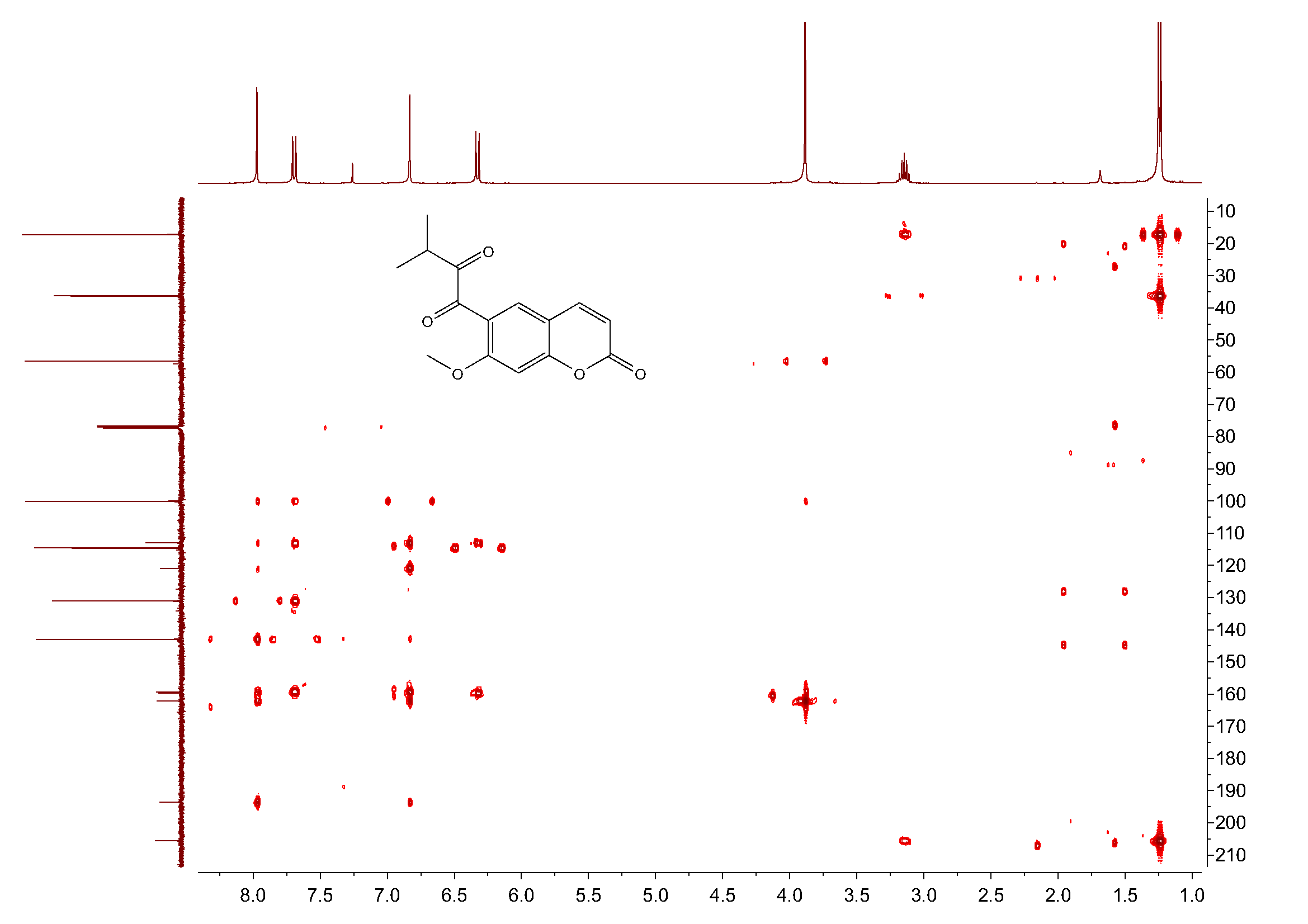


**S11.** ^1^H NMR spectrum (400 MHz, CDCl_3_) of 6-hydroxycoumurrayin (**4**)


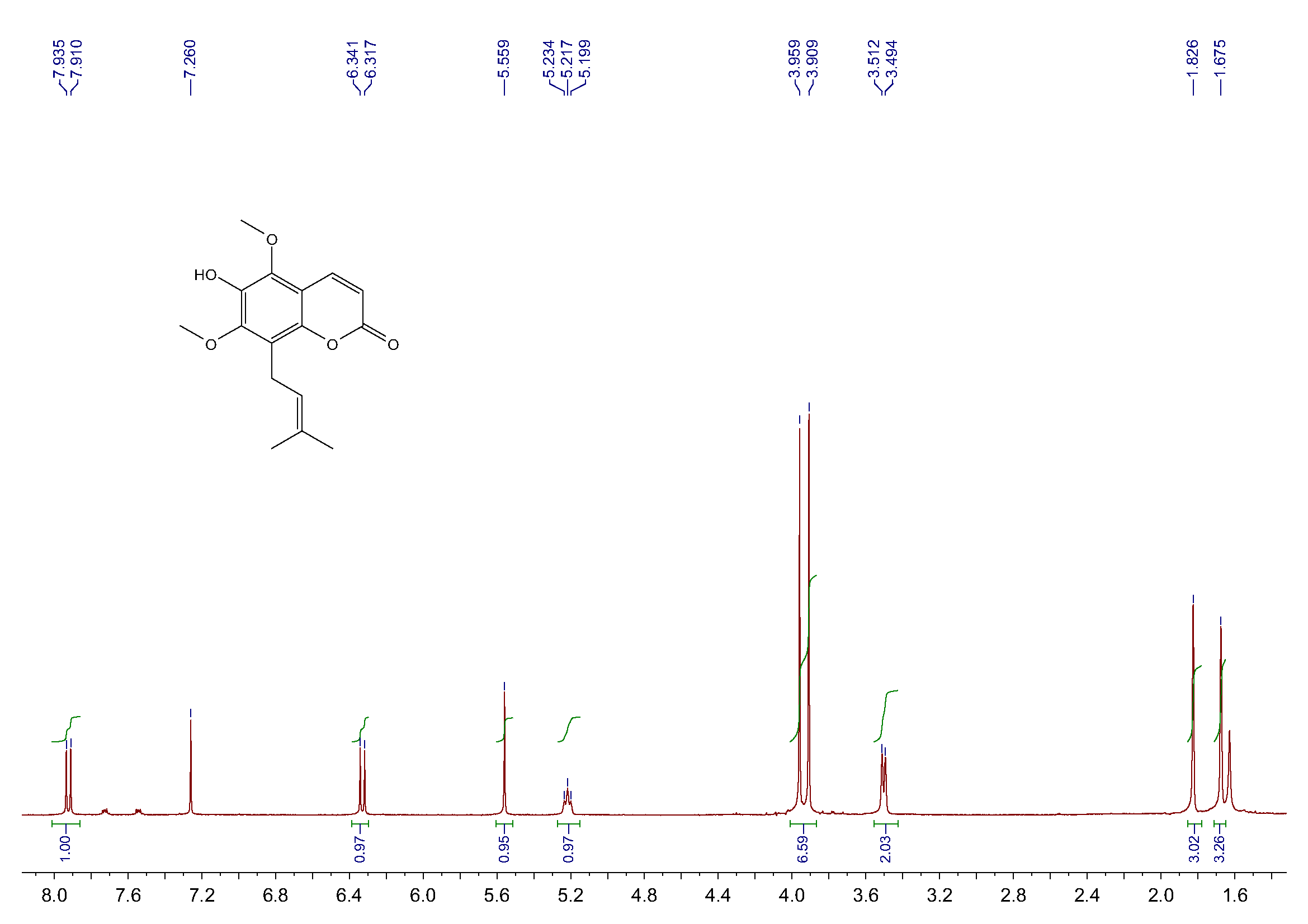


**S12.** ^13^C NMR spectrum (150 MHz, CDCl_3_) of 6-hydroxycoumurrayin (**4**)


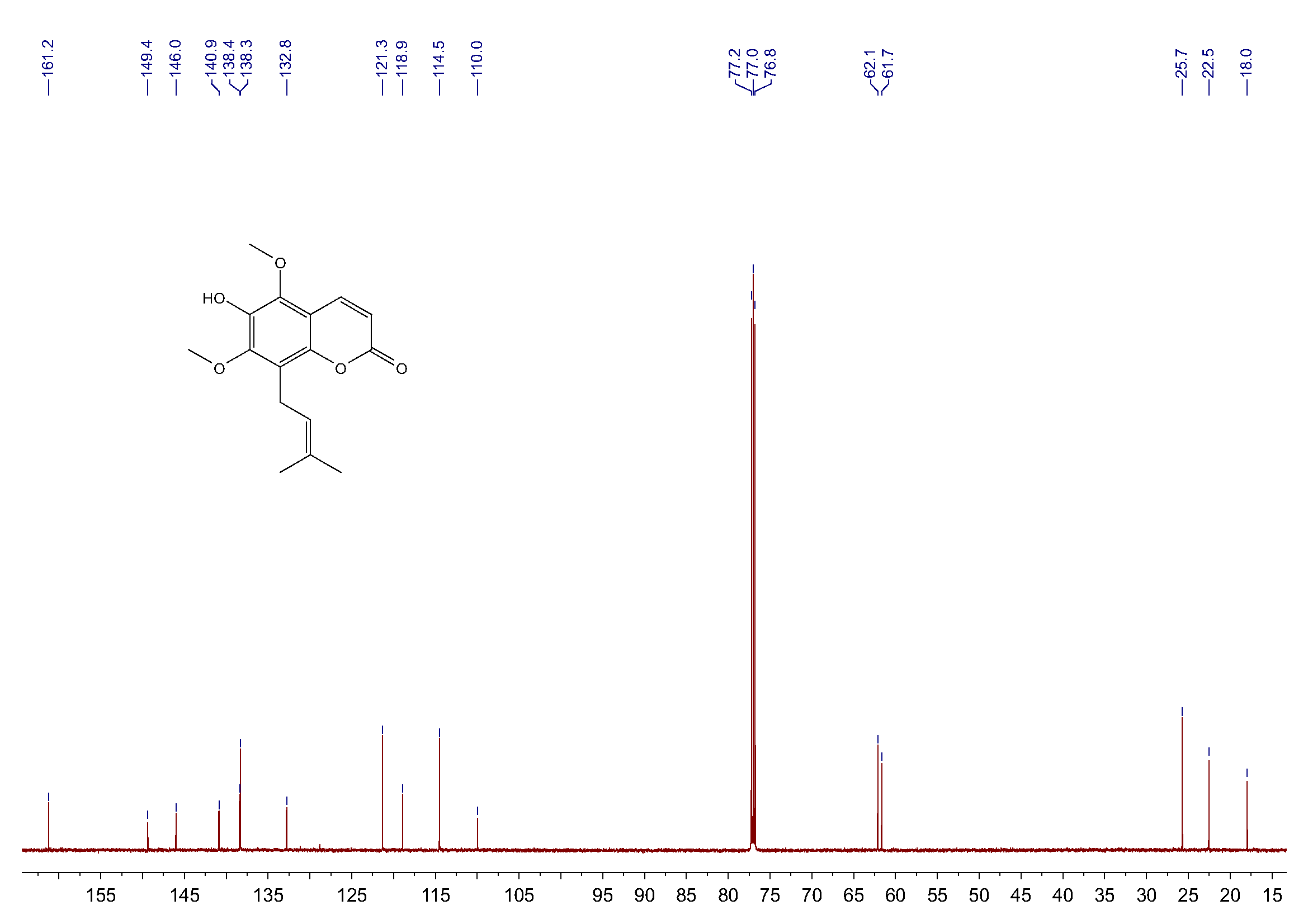


**S13.** HMBC spectrum (600 MHz, CDCl_3_) of 6-hydroxycoumurrayin (**4**)


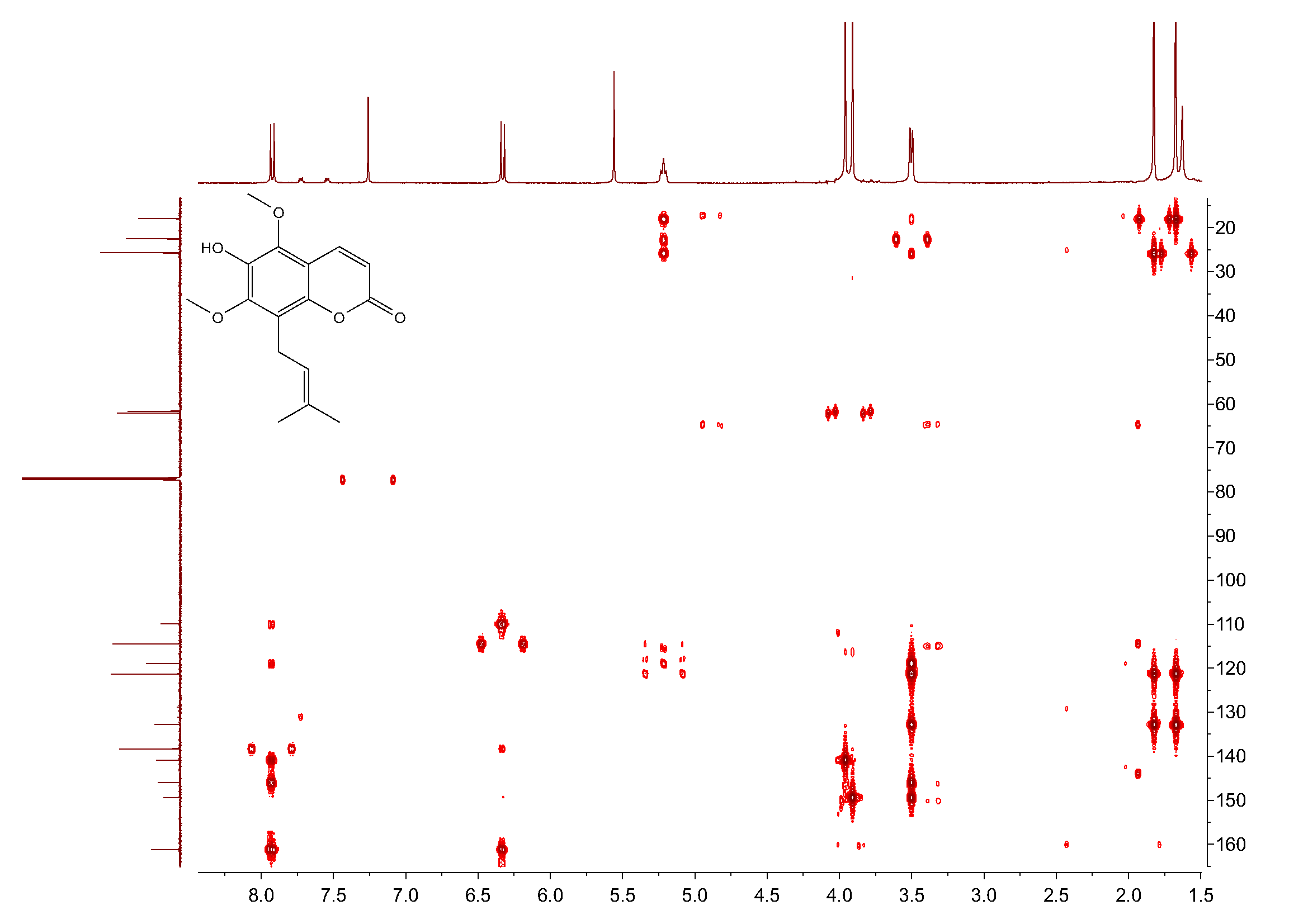

Supplement: Supplementary file 1 — Supplementary material 1 (DOCX 541 kb) [file 13659_2016_107_MOESM1_ESM.docx]
